# Klinefelter syndrome and other sex chromosomal aneuploidies

**DOI:** 10.1186/1750-1172-1-42

**Published:** 2006-10-24

**Authors:** Jeannie Visootsak, John M Graham

**Affiliations:** 1 Department of Human Genetics, Emory University School of Medicine, Atlanta, GA 30033, USA; 2 Medical Genetics Institute, Steven Spielberg Pediatric Research Center, Department of Pediatrics, Cedars-Sinai Medical Center, David Geffen School of Medicine at UCLA, Los Angeles, CA, USA

## Abstract

The term Klinefelter syndrome (KS) describes a group of chromosomal disorder in which there is at least one extra X chromosome to a normal male karyotype, 46,XY. XXY aneuploidy is the most common disorder of sex chromosomes in humans, with prevalence of one in 500 males. Other sex chromosomal aneuploidies have also been described, although they are much less frequent, with 48,XXYY and 48,XXXY being present in 1 per 17,000 to 1 per 50,000 male births. The incidence of 49,XXXXY is 1 per 85,000 to 100,000 male births. In addition, 46,XX males also exist and it is caused by translocation of Y material including sex determining region (SRY) to the X chromosome during paternal meiosis. Formal cytogenetic analysis is necessary to make a definite diagnosis, and more obvious differences in physical features tend to be associated with increasing numbers of sex chromosomes. If the diagnosis is not made prenatally, 47,XXY males may present with a variety of subtle clinical signs that are age-related. In infancy, males with 47,XXY may have chromosomal evaluations done for hypospadias, small phallus or cryptorchidism, developmental delay. The school-aged child may present with language delay, learning disabilities, or behavioral problems. The older child or adolescent may be discovered during an endocrine evaluation for delayed or incomplete pubertal development with eunuchoid body habitus, gynecomastia, and small testes. Adults are often evaluated for infertility or breast malignancy. Androgen replacement therapy should begin at puberty, around age 12 years, in increasing dosage sufficient to maintain age appropriate serum concentrations of testosterone, estradiol, follicle stimulating hormone (FSH), and luteinizing hormone (LH). The effects on physical and cognitive development increase with the number of extra Xs, and each extra X is associated with an intelligence quotient (IQ) decrease of approximately 15–16 points, with language most affected, particularly expressive language skills.

## Disease name and synonyms

Klinefelter syndrome (KS) – XXY male – XX male – XXYY male – XXXY male – XXXXY male

## Definition

The term Klinefelter syndrome describes a group of chromosomal disorder in which there is at least one extra X chromosome added to a normal male karyotype, 46,XY. The classic form is the most common chromosomal disorder, in which there is one extra X chromosome resulting in the karyotype of 47,XXY. As more individuals suspected of having Klinefelter syndrome had chromosome studies done, other karyotypes were sometimes observed, such as 48,XXYY; 48,XXXY and 49,XXXXY.

## Background

In 1942, Dr Harry Klinefelter published a report on nine men with a constellation of features: testicular dysgenesis, elevated urinary gonadotropins, microorchidism, eunuchoidism, azoospermia, and gynecomastia [1]. It was believed to be an endocrine disorder of unknown etiology, until 1959, when Jacobs *et al*. recognized that Klinefelter syndrome was a chromosomal disorder in which there is an extra X chromosome resulting in the karyotype of 47,XXY [2]. During the early 1970's, a number of centers began screening newborns for sex chromosomal abnormalities, because there was a need to obtain accurate information about childhood development in this condition [3]. Previous studies of XXY individuals were extremely biased toward more severely affected individuals, since these patients were drawn largely from mental or penal settings where large numbers of men could be screened. These earlier studies implied a risk for mental deficiency and behavioral problems. As prospective, unbiased studied have reported their results in recent years, it has become clear that most XXY boys demonstrate reductions in speech and language abilities which are correlated with decreased reading and spelling achievement [4]. Most, but not all XXY males, are infertile with small testicles, increased numbers of Leydig cells, tubular sclerosis, and interstitial fibrosis of varying degrees [5]. Their ejaculate is usually azoospermic, and levels of testosterone are typically low to low-normal.

## Diagnostic criteria

Formal cytogenetic analysis is necessary to make a definite diagnosis, and more obvious differences in physical features tend to be associated with increasing numbers of sex chromosomes. Chromosome analysis on lymphocytes from peripheral blood, or on amniocytes or chorionic villi from prenatal specimens is used to make this diagnosis.

If the diagnosis is not made prenatally, 47,XXY males may present with a variety of subtle clinical signs that are age-related. In infancy, males with 47,XXY may have chromosomal evaluations done for hypospadias, small phallus or cryptorchidism [6]. In the toddler years, boys may present with developmental delay, especially with expressive language skills [7]. The school-aged child may present with language delay, learning disabilities, or behavioral/social problems [7]. The older child or adolescent may be discovered during an endocrine evaluation for delayed or incomplete pubertal development with eunuchoid body habitus, gynecomastia, and small testes [8]. Adults are often evaluated for infertility or breast malignancy [9].

## Epidemiology

XXY aneuploidy is the most common disorder of sex chromosomes in humans, with a prevalence of one in 500 males [3]. Other sex chromosomal aneuploidies are much less frequent with 48,XXYY and 48,XXXY being present in 1 per 17,000 to 1 per 50,000 male births. The incidence of 49,XXXXY is 1 per 85,000 to 100,000 male births [10]. Cases of 46,XX males have also been reported.

## Clinical description

### A. Physical characteristics

Boys with 47,XXY have variable phenotypic characteristics and do not have obvious facial dysmorphology; thus, they are indistinguishable from other boys with normal karyotypes [6]. Small testicular size is the only consistent physical feature in 47,XXY. The presence of gynecomastia and other findings of eunuchoid body habitus and sparse body hair are variable.

#### Growth/Stature

Infants and children have normal heights, weights, and head circumferences. The increase in height is most significant between ages 5 and 8 and results in the mean final height of 179.2 + 6.2 cm. Affected individuals have longer arms and legs [11,12].

#### Secondary sexual characteristics

Many 47,XXY boys appear to enter puberty normally with a tendency for testosterone concentrations to decline at late adolescence and early adulthood. With a decrease in androgen production, secondary sexual characteristics do not completely develop, and features of eunuchoidism and gynecomastia can develop. This also results in sparse facial, body, and sexual hair [8]. The reported incidence of gynecomastia in Klinefelter syndrome varies widely from 56% to 88% [5].

#### Gonads

Features that are constant in 47,XXY males are small, soft testes with elevated gonadotropins. Testicular volume is typically less than 10 ml in postpubertal 47,XXY individuals [5].

#### Fertility

Although most patients with Klinefelter syndrome are infertile, there have been a few patients with reports of pregnancy without assisted medical technology, typically in mosaic cases. With the introduction of intracytoplasmic sperm injection, which involves the use of sperm extraction from deep within the testicles of patients with nonmosaic Klinefelter syndrome, some XXY men will have an increased chance of fathering a child [9,13-15]. A study of 42 men with Klinefelter syndrome revealed that the sperm retrieval rate was 72% per testicular sperm extraction attempt, and 69% (29 of 42 men) had adequate sperm found using intracytoplasmic sperm injection. Thus, testicular sperm extraction and intracytoplasmic sperm injection may be considered in males with azoospermia and Klinefelter syndrome [15].

### B. Psychological characteristics

#### Intelligence

A wide range of intelligence quotient (IQ) has been noted and extends from well below average to well above average. Based on the Wechsler Intelligence Test, Verbal IQ is usually lower than Performance IQ. Most of the differences between Verbal IQ and Performance IQ appear to relate to deficits in verbal abilities and to decreased auditory memory and processing [16].

#### Language development

Several longitudinal studies of males with 47,XXY have revealed a tendency for language deficits that often causes academic difficulties during the school years. Most 47,XXY boys have a lag in language skills with mildly delayed expression of single words. These individuals also demonstrate that the production of expressive language is affected more than that of comprehension or receptive skills [4]. The pattern of deficits includes problems in understanding of complex grammatical constructions, problems in oral language production, and deficits in morphology, word retrieval abilities, and oral narrative construction. The variability of their speech and language deficits is reflected in the lower mean verbal scales scores being significantly lower than performance scale scores [4].

#### Behavior and personality

The personalities of 47,XXY males are variable. One study characterized 47,XXY males as timid, immature, and reserved, with difficulty relating to their peer group, whereas other studies described 47,XXY subjects as friendly, kind, helpful, and relates well with other people. Most are described to be quiet, sensitive, and unassertive. The majority of 47,XXY males rate themselves as more sensitive, apprehensive, and insecure than their peers. An increased incidence of anxiety, depression, and substance abuse is reported in adolescents with 47,XXY [17]. The language difficulty experienced by these males possibly contributes to the challenges in behavioral and social domains [18].

### C. Complications

• Risk of acquiring breast carcinoma in 47,XXY is relatively increased, with relative risk exceeding 200 times [19]. The cause may result from the estradiol to testosterone ratio being severalfold higher than that of karyotypically normal men or possibly due to an increased peripheral conversion of testosterone to estradiol in men with Klinefelter syndrome [19].

• Associated endocrine complications include diabetes mellitus, hypothyroidism, and hypoparathyrodism [20].

• Autoimmune diseases, such as systemic lupus erythematosus, Sjogren syndrome, and rheumatoid arthritis, are more common in Klinefelter syndrome, with frequencies similar to those found in 46,XX females.

• Development of varicose veins and leg ulcers may result from venous stasis [21].

• Decreased bone density occurs in 25% of patients with Klinefelter syndrome, possibly reflecting the impact of decreased bone formation, increased bone resorption and/or hypogonadism [22].

## Etiology

The extra X chromosome in 47,XXY results sporadically from either meiotic nondisjunction where a chromosome fails to separate during the first or second division of gametogenesis or from mitotic nondisjunction in the developing zygote. The likelihood of X chromosome nondisjunction increases with advancing maternal age.

The effects on physical and mental development increase with the number of extra Xs, and each extra X is associated with an IQ decrease of approximately 15–16 points, with language most affected, particularly expressive skills [10].

## Diagnostic testing

A karyotype analysis of peripheral blood is the gold standard.

Elevated follicle stimulating hormone (FSH), luteinizing hormone (LH) and estradiol, and low to low-normal testosterone level without testosterone therapy.

Urinary gonadotropins are increased due to abnormal Leydig cell function.

## Differential diagnosis

The physical manifestations of Klinefelter syndrome are often variable. When the following features are present in an undiagnosed male, a karyotype analysis may be indicated:

• Small testes

• Infertility

• Gynecomastia

• Long legs and arms

• Developmental delay

• Speech and language deficits

• Learning disabilities or academic issues

• Psychosocial difficulties

• Behavioral issues

Other causes of hypogonadism need to be considered, such as Kallmann syndrome.

## Genetic counseling

The recurrence risk is not increased above that of the general population. There is no evidence to suggest that a chromosomal nondisjunction process is likely to repeat itself in a particular family.

## Antenatal diagnosis

Klinefelter syndrome can be detected prenatally by amniocentesis and cytogenetic amniotic fluid. Parents should be counseled based on recent prospective and unbiased information.

## Management

### Testosterone treatment

Androgen replacement therapy should begin at puberty, around age 12 years, in increasing dosage sufficient to maintain age appropriate serum concentrations of testosterone, estradiol, FSH, and LH. Androgen replacement promotes normalization of body proportions or development of normal secondary sex characteristics, but does not treat infertility, gynecomastia, and small testes. Testosterone replacement also results in general improvement in behavior and work performance [23]. Testosterone also has beneficial long-term effects that might reduce the risk of osteoporosis, autoimmune disease, and breast cancer [24].

### Speech therapy

Early identification and anticipatory guidance are important in boys with 47,XXY. Early speech/language therapy is particularly essential in helping the child to develop skills in the understanding and production of more complex language.

### Physical therapy

Physical therapy should be considered for boys who have hypotonia or delayed in gross motor skills which may impact the muscle tone, balance, and coordination.

### Occupational therapy

If boys with 47,XXY have fine motor dyspraxia, occupational therapy should be recommended. In addition, an occupational therapist may benefit infants with 47,XXY who have feeding problems or difficulty with latching on or sucking.

### Educational services

Males with 47,XXY should receive a comprehensive psychoeducational evaluation to assess their areas of strengths and weaknesses. The information obtain from this evaluation may be helpful in planning appropriate resources and classroom placement. Consultation with a developmental-behavioral pediatrician is suggested.

## Other sex chromosomal aneuploidies

### 48,XXYY

Males with 48,XXYY are often tall, with an adult height above 6 feet. They may have an eunuchoid habitus with long legs, sparse body hair, small testicles and penis, hypergonadotropic hypogonadism, and gynecomastia. Peripheral vascular disease may result in leg ulcers and varicosities. Their IQ level is in the range of 60–80, with delayed speech and they are at risk for academic, behavioral, and social deficits. They are usually shy but can be aggressive and impulsive [10,25,26]. In a study of 16 males with 48,XXYY compared to 9 males with 47,XXY between the ages of 5 and 20, findings indicate that 48,XXYY males have verbal and full scale IQ's significantly lower than males with 47,XXY [27]. 48,XXYY males are also prone to have problems with hyperactivity, aggression, conduct, and depression compared to males with 47,XXY. Their mean scores in these areas are in the clinically significant range and males with 47,XXY have scores in the average range. Furthermore, 48,XXYY males have significantly lower adaptive functioning than males with 47,XXY [27].

### 48,XXXY

Males with 48,XXXY chromosome karyotype can be average or tall stature with ocular hypertelorism, flat nasal bridge, radioulnar synostosis, fifth-finger clinodactyly, and small penis and testicles with hypergonadotropic hypogonadism. Their IQs are usually between 40 and 60, with severely delayed speech. Their behavior is often immature and consistent with their IQ level, and they are typically described as passive, cooperative, and not particularly aggressive [10,25,26].

### 49,XXXXY

Males with 49,XXXXY are severely affected. They manifest microcephaly with short stature, ocular hypertelorism, flat nasal bridge, and upslanting palpebral fissures. They may also have a bifid uvula, cleft palate, heart defect (usually patent ductus arteriosus), radioulnar synostosis, genu valgum, pes cavus, fifth-finger clinodactyly, hypotonia with lax joints, and small genitalia with hypergonadotropic hypogonadism. Their IQ ranges between 20 to 60. They tend to be shy and friendly, with occasional irritability and temper tantrums, low frustration tolerance, and difficulty changing routines [10,25,26].

### 46,XX male

46,XX male chromosomal karyotype is caused by translocation of Y material including sex determining region (SRY) to the X chromosome during paternal meiosis [28]. Existence of sexual determining factor on X chromosome leads to normal male sexual development. Males with 46,XX typically have normal external genital development, but hypospadia or cryptochidism may be seen [29]. In addition, males with 46,XX also have decrease testosterone level with high levels of LH and FSH and infertility may be present [29].
